# Microbial Surface Confined Growth Strategy for the Synthesis of Highly Loaded NiCoP Nanoparticles with Hollow Derived Carbon Shells for Sodium Ion Capture

**DOI:** 10.1002/advs.202407616

**Published:** 2024-10-28

**Authors:** Jianhua Yuan, Tianxiao Sun, Jinfeng Chen, Runhong Zhou, Jianglin Cao, Fei Yu, Liqing Li, Xiumin Zhong, Jie Ma

**Affiliations:** ^1^ School of Chemistry and Chemical Engineering Jiangxi University of Science and Technology Ganzhou 341000 P. R. China; ^2^ Research Center for Environmental Functional Materials College of Environmental Science and Engineering Tongji University 1239 Siping Road Shanghai 200092 P. R. China; ^3^ School of Civil Engineering Kashi University Kashi 844000 P. R. China; ^4^ Helmholtz‐Zentrum Berlin für Materialien und Energie GmbH Hahn‐Meitner‐Platz 1 14109 Berlin Germany; ^5^ College of Oceanography and Ecological Science Shanghai Ocean University Shanghai 201306 P. R. China

**Keywords:** highly loaded, hollow carbon shell, sodium ion capture, surface confined growth, transition metal phosphides

## Abstract

NiCoP is considered to be a very promising material for sodium ion (Na^+^) capturing, however, the volume expansion and poor cyclic stability of NiCoP during the storage limit its application. In response to these limitations, Finite element simulations are used to help in the rational design of the NiCoP structure. A novel microbial surface confined growth strategy is employed to synthesize highly loaded NiCoP nanoparticles (NiCoP NPs) supported on hollow derived carbon shells (NPC), constructing a stable composite structure known as NiCoP@NPC. The highly loaded and uniformly dispersed NiCoP NPs are anchored in‐situ and fully exposed, enabling enhanced electron and ion transport efficiency and thereby boosting pseudocapacitance. The NPC from yeast played a crucial role in mitigating the volume expansion of NiCoP NPs, thereby enhancing the structural stability of the electrode. Consequently, NiCoP@NPC demonstrated a high Na^+^ storage capacity of 59.70 ± 1.51 mg g^−1^ at 1.6 V and maintained good cycling stability, retaining over 73.3% of its capacity after 80 cycles at 1.6 V. Scanning transmission X‐ray microscopy (STXM) analysis confirmed the reversible conversion reaction mechanism and the robust structure of NiCoP@NPC before and after the reaction; Density function theory (DFT) and electrochemical quartz crystal microbalance (EQCM‐D) further confirmed that the structural design of NiCoP@NPC promoted electron transport, Na^+^ adsorption as well as improved cycling stability. This study is intended to provide a new idea for the in‐situ confined synthesis of metal phosphides electrodes with stable performance and structure.

## Graphical abstract

A novel microbial surface confined growth strategy is employed to synthesize highly loaded NiCoP nanoparticles supported on hollow derived carbon shells, which enables enhanced electron and ion transport efficiency and boosts pseudocapacitance.

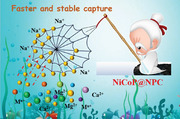

## 1 Introduction

Electrochemical Na^+^ storage technology has attracted significant attention for its flexible operation, low energy consumption, and environmental friendliness. Electrodes play a crucial role in determining its performance.^[^ ^1^ ^]^ Therefore, the design and construction of electrodes with minimal volume change and stable cycling performance have been an important part of this technology.^[^ ^2^ ^]^ Various types of electrodes, including activated carbon (AC), metal oxides, metal sulfides, and metal phosphides (TMPs) have been explored. TMPs like CoP, Ni_2_P, ZnNiP, and NiCoP are becoming a preferred option due to its high theoretical capacity, numerous electron orbitals, and low intercalation potentials.^[^ ^3^ ^]^ However, during the repeated process of Na^+^ embedding and detaching, due to the large ionic radius of the Na^+^ (0.102 nm), TMPs undergo volume changes caused by lattice variations, which leads to particle fragmentation and cleavage, resulting in a rapid decline in capacity and cycle instability.^[^ ^4^ ^]^ Furthermore, the electrodes face higher stability demands in aqueous conditions.^[^ ^5^ ^]^ Therefore, a strategic structural design is essential to alleviate stress concentration and mitigate the drastic volume changes of TMPs electrodes during the cycling process.^[^ ^6^ ^]^

NiCoP is considered a highly promising Na^+^ capturing material for TMPs.^[^ ^7^ ^]^ However, NiCoP also faces challenges such as large volume variation and cycling instability during surface pseudocapacitance and conversion reaction.^[^ ^8^ ^]^ To address these issues, several effective strategies have been proposed, including in‐situ preparation of small‐sized NiCoP particles, anchoring and dispersion of NiCoP NPs, and composite with different types of carbon materials.^[^ ^9^ ^]^ In‐situ preparation of small‐sized NiCoP particles is a common strategy,^[^ ^10^ ^]^ e.g., Zhang et al. deposited NiCoP particles with diameters less than 20 nm onto porous carbon substrates, and observed a high discharge capacity of 1462.7 mAh g^−1^ at 0.1 C.^[^ ^11^ ^]^ However, while the reduction in particle size enhanced reaction rates and specific capacity, it did not yield satisfactory improvements in electrical conductivity and cycling performance.^[^ ^12^ ^]^ Anchoring and dispersing NiCoP NPs can enhance electron transport efficiency and increase the availability of redox‐active sites,^[^ ^13^ ^]^ according to Pang et al. The researchers demonstrated this by synthesizing NiCoP NPs on a bimetallic‐organic framework, leading to a specific capacitance of 525 F g^−1^ at 0.5 A g^−1^.^[^ ^14^ ^]^ However, the aggregation tendency of high surface energy NPs can lead to electrode activity degradation and reduced cycling performance.^[^ ^15^ ^]^ Additionally, compositing NiCoP NPs with graphene, carbon nanotubes, carbon nanofibers, etc. has proven effective, however, it tends to lead to the reduction of specific capacitance and power density when the carbon content is high.^[^ ^16^ ^]^ Thus, a rational structural design is essential for achieving high electrochemical performance and cycling capability in NiCoP electrodes.^[^ ^17^ ^]^

Surface confined growth is a compelling approach for controlling the growth of NPs on specific surfaces.^[^ ^18^ ^]^ Nanomaterials with distinctive physicochemical properties, such as nanowires, NPs, and nanosheets, are prepared on carrier surfaces by carefully selecting and adjusting reaction conditions.^[^ ^19^ ^]^ These materials are widely used in catalysis, oxidation, and synthetic transformations.^[^ ^20^ ^]^ Achieving highly selective growth on specific crystal faces or orientations is possible through the use of surface modifiers or the creation of nucleation sites.^[^ ^21^ ^]^ Various methods, including employing carrier templates, manipulating reaction conditions, and incorporating surface modifiers, can facilitate surface‐limited growth.^[^ ^22^ ^]^ However, more novel methods need to be further investigated.^[^ ^23^ ^]^ Yeast cells are degradable, low‐cost, and readily available microorganisms with surfaces rich in charge and active sites (amino, carboxyl, hydroxyl, amide groups, etc.).^[^ ^24^ ^]^ These characteristics provide numerous nucleation centers for interactions with metal ions (M^+^), enabling the control of NPs nucleation and growth. Moreover, the loading isolation mode on the yeast surface effectively prevents NPs agglomeration.^[^ ^25^ ^]^ Moreover, the uptake of M^+^ by yeast cells, involving surface adsorption and metabolic translocation, is crucial in inhibiting the excess assembly and growth of NPs.^[^ ^26^ ^]^ Consequently, leveraging the highly active and proportional surface‐active sites of yeast cells for in‐situ confined growth presents a promising strategy for achieving highly loaded and uniformly dispersed NiCoP NPs.^[^ ^27^ ^]^ However, research on microbial‐coupled surface confined growth strategies remains scarce, and the precise design of NiCoP composite structures poses challenges. Further exploration is warranted to elucidate the Na^+^ capture mechanism of these composite structures.

Having considered the above, the stress evolution of NiCoP NPs and composite structures during the electrochemical driving process was simulated by establishing a finite element model. Guided by an effective stress management model, a yeast surface‐limited growth approach was implemented to successfully prepare a hollow composite structure, denoted as NiCoP@NPC. This innovative structure comprises highly loaded and uniformly dispersed NiCoP NPs within NPC, marking its pioneering application in Na⁺ storage. This precisely designed structure offers distinct advantages. The highly loaded NiCoP NPs can significantly promote the diffusion of Na^+^ and effectively improve the kinetics of the electrode. The NPC acts as a supportive structure and elastic substrate, consequently bolstering the stability of the NiCoP framework and accommodating volume expansion. Therefore, the prepared NiCoP@NPC exhibits excellent Na^+^ storage and cycling stability performance, which can be attributed to the confined growth of NiCoP NPs containing abundant active sites and short Na^+^ diffusion paths, as well as the coupling of the pseudocapacitance of NiCoP NPs to the role of the NPC's Electrical double‐layer (EDL).

## 2 Results and Discussion

**Figure 1:**
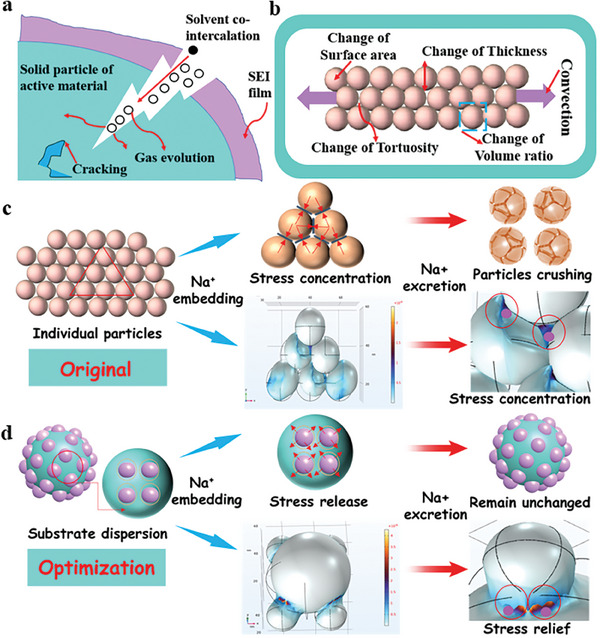
a) Internal changes in the electrode during ion embedding and removal. b) Schematic representation of model of volume expansion effect. Progressive stress management strategy and finite element simulation of displacement and volume expansion of NiCoP NPs under particle accumulation c) and NiCoP@NPC prepared after optimization strategy d).

During the charging and discharging process, As shown in **Figure** **1**a, Na^+^ in are consistently absorbed and released in the negative electrode, resulting in the expansion of its volume.^[^ ^28^ ^]^ This volume change is primarily induced by internal stresses, with the added factor of localized gravitational forces leading to the fracturing of negative electrode particles. In addition, co‐insertion of solutes with Na^+^ also causes the particles to rupture, especially the gas generated during co‐insertion. The analysis revealed that electrodes typically undergo changes in thickness, volume fraction, electrochemical surface area, convection, and tortuosity during ion embedding and removal^[^ ^29^ ^]^ (Figure 1b).

The original and optimized NiCoP are modeled separately. According to the classical Cahn‐Hilliard ion diffusion theory,^[^ ^30^ ^]^ analyzing the stress of stacked NPs during Na^+^ storage shows that the contact between particles is a point‐to‐point contact, and this type of contact will expand due to the excessive stress gradient, which can easily lead to particle cracking (Figure 1c). In order to overcome this same defect in NiCoP NPs, a microbial surface confined growth strategy for the synthesis of highly loaded NiCoP NPs was proposed. This strategy focuses on changing the contact mode of the NPs and increasing the surface contact by fixing the NiCoP NPs at the NPC. During the expansion process, the NPC are subjected to uniform stresses in different directions by cushioning the soft interface of the NPCs, which reduces the problems of volume expansion and cracking caused by stress concentration (Figure 1d). In order to verify the correctness of the theory, the finite element simulations were carried out (Figure S1a‐b, Supporting Information) and found that under the same displacement conditions, the deformation of the stacked NiCoP NPs mainly occurs in the ions themselves, while stress concentration and displacement distortion occurs at the location of the point‐to‐point contact (Figure 1c; Figure S2a‐b, Supporting Information). Additionally, the simulations results show that NiCoP@NPC electrode displayed a smaller stress value of 1.18 × 10^5^ MPa in comparison with the NiCoP NPs electrodes, which had a stress value of 1.06 × 10^6^ MPa, with no visible stress concentration on its surface (Figure 1d; Figure S2c‐d, Supporting Information). This indicated that although the overall volume change of the complexes occurred slightly, the NiCoP NPs did not produce obvious deformation. Thus, further illustrating this stress management strategy plays a significant role in alleviating stress concentration and volume expansion. By fixing the NiCoP NPs at the NPC's surface and subjecting them to uniform stresses through a buffer management strategy, the stress concentration and volume expansion issues have been effectively mitigated.

**Figure 2:**
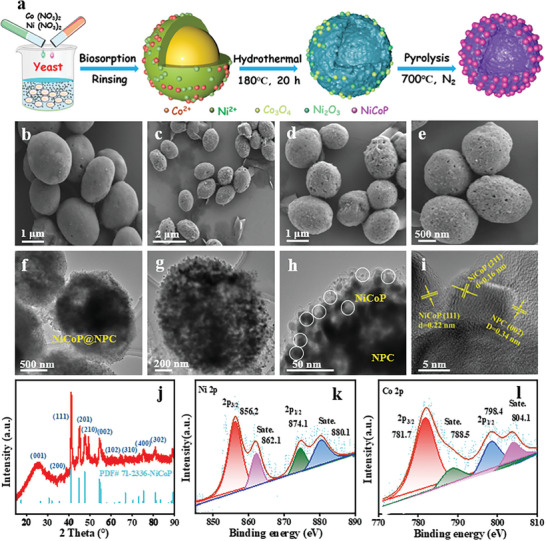
a) Schematic illustration of the synthesis of NiCoP@NPC. SEM image of NPC b) and NiCoP@NPC c‐e). TEM images of NiCoP@NPC f, g, h, i). j) XRD pattern of the NiCoP@NPC. High‐resolution XPS spectra of Ni 2p k) and Co 2p l) are depicted.

**Figure** **2**a illustrates the fabrication process of NiCoP@NPC. First, *Saccharomycete* Yeasts were cultured using shaking flasks as a biological carrier and nitrogen/carbon sources (Figure S3a, Supporting Information). Next, the obtained strains were submerged in the mixed solution of cobalt nitrate and nickel nitrate for microbial assembly and metabolism (Figure S3b‐c, Supporting Information). During this process, the surface of *Saccharomycete* contained various functional groups can “in situ‐anchor” of charged ions such as Ni^2+^ and Co^2+^. Finally, the NiCoP@NPC was prepared through hydrothermal, filtering, cold‐drying, and calcination (Figure S3d‐f, Supporting Information). During hydrothermal and high‐temperature calcination, the yeast's surface graphitized to create a shell layer. Simultaneously, the yeast's structure contracted as amorphous carbon formed inside, leading to the development of a hollow structure. After adsorption on the yeast surface, Ni^2+^ and Co^2+^ react and transform at high temperatures with elemental phosphorus diffusing outward from the cell, completing in situ confined growth of the NiCoP NPs. It should be emphasized that during the synthesis process, only the phosphorus contained in the yeast was used as a phosphorus source, and the samples were obtained by in situ phosphorylation without the addition of any additional phosphorus source. Furthermore, NPC and NiCoP as contrast to NiCoP@NPC were synthesized.

The morphologies and structure of *Saccharomycete* and NiCoP@NPC were examined with scanning electron microscopy (SEM) and transmission electron microscopy (TEM). As shown in Figure 2b, *Saccharomycete* had a spherical structure, ≈2–3 µm in diameter, with a smooth surface. The average nitrogen and phosphorus composition of yeast cells was 7.5‐10% and 1.6‐3.5%, respectively. Which is the basis for in‐situ phosphorylation and in‐situ doping. In addition, the NPC has good electrical conductivity, which facilitates electron conduction/transfer, and also improves the dispersion of the loaded NPs. After calcination, the NPC is a spherical or ellipsoidal hollow structure, while the NiCoP NPs are uniformly immobilized on the surface of the NPC, so more active sites are exposed and the pseudocapacitance is enhanced, therefore improving the Na^+^ adsorption properties (Figure 2c‐e). The hollow structure enhances electrode utilization efficiency by reducing dead volume, ensuring better contact with the electrolyte, and increasing the number of active sites, thus improving ion storage capacity.^[^ ^18a^ ^]^ Elemental mapping analyses (Figure S4a‐f, Supporting Information) revealed homogeneous distribution of N, C, P, Ni, and Co elements of the sample, meanwhile, the ratio of Ni, Co, and P elements in NiCoP@NPC is basically 1:1:1, which further confirms that the synthesized samples are NiCoP NPs. The TEM analysis provided more detailed structures of the NiCoP@NPC. NiCoP NPs were observed as spherical particles anchored on the NPC surface (Figure 2f‐h). These findings demonstrate that *Saccharomycete* provides active sites for Ni^2+^, Co^2+^ and are well suited to derive hollow structures. High‐resolution TEM (HRTEM) images in Figure 2i reveal obvious lattice fringes of 0.16, 0.22, and 0.34 nm assigned to the NiCoP (111), NiCoP (113), and NPC (002), respectively, indicating successful formation of NiCoP that provides ample active interface and facilitates ion diffusion.

Analysis of the XRD results shows that peaks ≈24° are attributed to NPC. The diffraction peaks in XRD patterns of NiCoP@NPC can be indexed to NiCoP (PDF# 71–2336) (Figure 2j). The full XPS spectrum in Figure S6a (Supporting Information) confirms that NiCoP@NPC contains C, N, Co, and Ni elements. Pyridinic N, pyrrolic N, and graphitic N, observed after calcination, confirm introduction of nitrogen species by NPC samples (Figure S6b, Supporting Information).^[^ ^31^ ^]^ The C 1s spectrogram in Figure S6c (Supporting Information) shows there were three peaks located at 284.4, 284.9, and 286.3 eV, which can be assigned to C‐P, C‐C/C = N, and C = O configurations, respectively.^[^ ^32^ ^]^ As shown in Figure S5d (Supporting Information), peaks at 130.3, 129.5, and 133.7 eV are assigned to P 2p_1/2_, 2p_3/2_, and P‐O, respectively. The occurrence of P‐O may be attributed to PO_4_^3−^ or P_2_O_5_, and its high content is due to surface oxidation of NiCoP@NPC.^[^ ^33^ ^]^ XPS results confirmed the successful in‐situ doping of N and P species by NPC. It was shown that the N and P co‐doping had higher binding energy for Na^+^ compared to single‐component doping. Meanwhile, the atomic radius of P is larger than that of C atoms, and the doping of P will change the local structure of the carbon skeleton while fine‐tuning the local charge and self‐selected state of N‐doped C, thus improving the electrochemical activity.^[^ ^34^ ^]^ The existence of NiCoP@NPC was verified by Ni 2p (Figure 2k) and Co 2p (Figure 2l). Four peaks in the Ni 2p curve identified at 856.2, 874.1, 862.1, and 880.1 eV corresponded to the Ni 2p_3/2_, Ni 2p_1/2_, and two satellite configurations, respectively. Meanwhile, four peaks in the Co 2p curve identified at 781.7, 798.4, 788.5, and 804.1 eV corresponded to the Co 2p_3/2_, Co 2p_1/2_, and two satellite configurations, respectively.

Figure S5e (Supporting Information) shows the Raman spectra for NiCoP@NPC. The peaks at 1355.4 and 1575.7 cm^−1^ correspond to the D and G bands, respectively, indicating disordered and graphitic structures. The spectra can be fit into four bands (D1, D2, and G) using Gaussian numerical simulation. D2 peaks are considered to be a stretching vibration of the C‐C bond in an aliphatic or olefin‐like structure. The ID/IG ratio reflects the degree of disorder in carbon structures. The I_D1_/I_G_ ratio of 2.11 indicates that the material has a high degree of defects at the edges of the carbon layer. The D2 proportion calculated for NiCoP@NPC was 3.82%, suggesting a higher amount of intrinsic defects in the NPC structure.^[^ ^25^ ^]^ Raman results also confirmed that the N/P doping increased the number of defects, leading to more active sites for enhanced adsorption.^[^ ^35^ ^]^

Figure S5f (Supporting Information) shows the N_2_ absorption‐desorption isotherms and pore size distribution of NiCoP@NPC, which exhibits a high specific surface area of 64.75 m^2^ g^−1^. When yeast is treated with a hollow structure and then undergoes calcination, it results in the formation of numerous small pores in the surface layer due to the exclusion of gases. This favors the increase of specific surface area and the increase of pores. The presence of micropores and mesopores in NiCoP@NPC is evidenced by the type IV adsorption isotherm with a hysteresis loop.^[^ ^36^ ^]^ NPC's surface contains pores that serve as ion transport channels during desalination. Consequently, the loading of NiCoP on the NPC's surface increased its specific surface area, prevented NPs agglomeration, and enabled more active sites to be exposed, leading to improved Na^+^ storage performance.^[^ ^37^ ^]^ Thermogravimetric tests (TGA) were used to determine the NiCoP content in NiCoP@NPC (Figure S6, Supporting Information). By calculation, the volatiles and water of crystallization produced by the yeast were ≈5.60%, and the carbon content after calcination was ≈10.1%, and NiCoP achieved a high loading with a content of 84.3%, thus indicating that the surface confined growth strategy effectively improved the loading of NPs.

**Figure 3:**
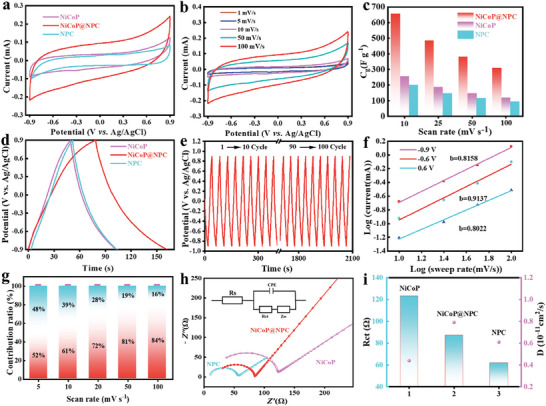
a) Cyclic voltammograms for NPC, NiCoP, and NiCoP@NPC at scan rate of 50 mV s^−1^. b) CV curves of the NiCoP@NPC at different scan rates. c) Gravimetric specific capacitances of NPC, NiCoP, and NiCoP@NPC at different scan rates. d) GCD profiles for NPC, NiCoP, and NiCoP@NPC at current density of 50 mA g^−1^. e) Long‐term GCD test of the NiCoP@NPC at 100 mA g^−1^. f) Calculation of b‐values based on CV curves. Normalized contribution ratio of capacitive capacitance at different scan rates g). h, i) Nyquist plots, R**_ct_** and Diffusion coefficient of different samples.

**Figure** **3**a illustrates the cyclic voltammetry (CV) curves of NPC, NiCoP, and NiCoP@NPC at different scan rate. All electrodes exhibited a rectangular‐like shape without redox peaks at various scan rates (Figure 3b; Figure S7a, Supporting Information), suggesting that the capacitive response is primarily attributed to the EDL capacitance.^[^ ^38^ ^]^ NiCoP@NPC exhibited a higher specific capacitance at varied scan rates compared to NPC and NiCoP. This is evident from the larger integral area of the CV curve (Figure 3c). The NiCoP@NPC electrode was found to be stable and reversible after 100 cycles at a scan rate of 1 mV s^−1^, as evidenced by highly overlapping CV shapes (Figure S7b, Supporting Information). This is probably due to the stable structure and synergistic promotion formed by NiCoP NPs and NPC. The electrochemical performance was further investigated by galvanostatic charge‐discharge (GCD) measurements. According to the results presented in Figure 3d, the NiCoP@NPC exhibited the longest charge/discharge time at a current density of 50 mA g^−1^, and this implied that it had the largest specific capacitance. The GCD curves of NiCoP@NPC showed no charging and discharging plateaus, indicative of EDL capacitance^[^ ^39^ ^]^ (Figure S7c, Supporting Information). GCD cycles were performed at a current density of 100 mA g^−1^, and the symmetry of each cycle was good, with a retention ratio of charging capacity after 100 cycles of 94.3% (Figure 3e). As shown in Figure 3f, the b‐values of the NiCoP@NPC electrode, calculated from the regions of the CV curves with potentials of −0.9 V, −0.6 V, and 0.6 V, were between 0.80 and 1.00, indicating that the dynamic process involves both diffusion and capacitance processes, and is mainly controlled by the capacitance contribution.^[^ ^20^ ^]^

The kinetic behavior and the source of capacitance contribution of the Na^+^ intercalation layer were also investigated to further examine the superiority of the designed structure. The capacitive contribution of the NiCoP@NPC electrode was found to increase from 52% at 50 mV s^−1^ to 84% at 100 mV s^−1^ (Figure 3f). The response was primarily controlled by capacitive effects, leading to high cycling stability and high‐rate performance. In addition, the Na^+^ reaction kinetics of the samples using electrochemical impedance spectroscopy (EIS) were evaluated. The Nyquist plots of NPC, NiCoP, and NiCoP@NPC exhibited a small semicircle and strong linearity (Figure 3h). According to the semicircles in the high‐frequency of EIS curves, the equivalent series resistance (R_s_) of NiCoP@NPC (35.50 Ω) is in the middle of all samples, larger than NPC (5.60 Ω) and smaller than NiCoP (42.50 Ω), which may be due to the the conductive property of NPC. The charge transfer resistance (R_ct_) of NiCoP@NPC (87.50 Ω) is smaller than that of NPC and NiCoP (R_ct_ = 62.3 and 123.4 Ω). This indicates that NiCoP@NPC has better electronic conductivity thanks to the 3D conductive skeleton formed by *Saccharomycete*. Moreover, the corresponding diffusion coefficient (*D*) values are calculated as shown in Figure 3i, it is obvious that the *D* of NiCoP@NPC (0.79 × 10^−11^ cm^2^ s^−1^) is higher than that of NPC (0.61×10^−11^ cm^2^ s^−1^) and NiCoP (0.44×10^−11^ cm^2^ s^−1^). The enhanced electrochemical performance of NiCoP@NPC was attributed to its large specific surface area of 64.75 m^2^ g^−1^ and hollow structure. The hollow structure provides unobstructed channels for electrolyte storage and Na^+^ transfer, facilitating full and close contact between the electrodes and electrolyte. Additionally, the outstanding redox reaction activity of NiCoP plays a crucial role in improving electrochemical reaction efficiency throughout the charge‐discharge process.^[^ ^40^ ^]^

**Figure 4:**
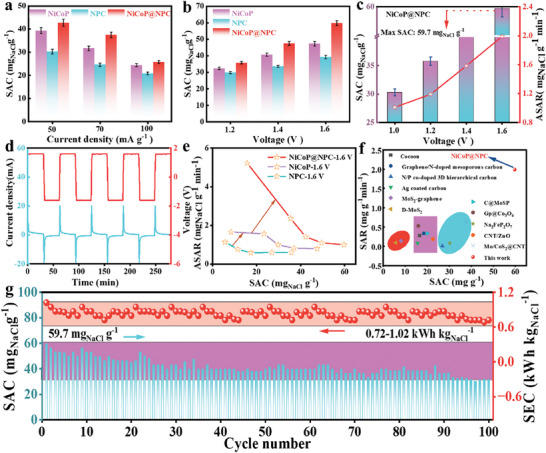
a) Na^+^ storage capacity at different specific currents of NPC, NiCoP, and NiCoP@NPC. b) Na^+^ storage capacity at different voltage of different samples. c) Na^+^ storage capacity and ASRA at different voltage of NiCoP@NPC. d) Graph of voltage and current density change versus time during desalination/regeneration of CDI process. e) Kim‐Yoon plots for NPC, NiCoP, and NiCoP@NPC electrodes. f) Comparison of Na^+^ storage capacity of other carbon‐based and TMPs based electrode materials. g) Long‐term cycling performance and corresponding specific energy consumption of NiCoP@NPC electrode.

NiCoP@NPC was used as the cathode and ACas the anode, with the schematic diagram of the CDI cell illustrated in Figure S8 (Supporting Information). The experiment tested the Na^+^ storage capacity and rate of NiCoP@NPC in 1000 mg L^−1^ of NaCl solution at 50, 70, and 100 mA g^−1^. As shown in **Figure** **4**a, the desalination capacity (SAC) and desalination rate (ASAR) of NiCoP@NPC exhibited opposite trends with the increase of current density. The maximum average ASAR was 1.512 mg g^−1^ min^−1^ and the SAC was 42.70 ± 1.51 mg g^−1^ at 50 mA g^−1^. The SAC of NPC, NiCoP, and NiCoP@NPC at 1.2, 1.4, and 1.6 V is presented in Figure 4b. NiCoP@NPC showed the highest SAC of 59.70 ± 1.51 mg g^−1^, which was ≈1.66 times that of NPC (35.80 ± 0.77 mg g^−1^) and 1.26 times that of NiCoP (45.70 ± 0.95 mg g^−1^). Meanwhile, when the cutoff voltage is 1.6 V, the conductivity decreases as shown in Figure S7d (Supporting Information), at which time the Na^+^ storage performance of NiCoP@NPC is optimal, and the voltage and current of the system during desalination remain stable (Figure 4c,d). The CDI Ragone plots, which merge SAC and ASAR, are shown in Figure 4e to assess the overall Na^+^ storage performance of various samples. It is evident that NiCoP@NPC exhibits a higher capacity and faster desalination rate, making it well‐suited for practical application.

To assess the CDI performance of NiCoP@NPC among the reported carbon and TMPs electrodes (Figure 4f),^[^ ^41^ ^]^ the relationship between SAC and SAR illustrated in the Kim‐Yoon plots was elaborated.^[^ ^42^ ^]^ NiCoP@NPC exhibits much higher SAC and SAR than any other electrode, as shown in Table S1 (Supporting Information). In the long‐cycle CDI experiments of NiCoP@NPC conducted with 1.60 V, and 1000 mg L^−1^ NaCl solution, the SAC of the first 25 cycles fluctuated up and down, while the SAC of the remaining cycles remained stable, as depicted in Figure 4g. After 80 cycles, the capacity retention rate was 73.3%, and after 100 cycles, the capacity retention rate was 52.4%. Reasons for the reduced cycling performance of the electrodes may include depletion of the NiCoP active material, electrode pulverization, and structural changes. The SEC per cycle of NiCoP@NPC was calculated to range from 85 to 92 kJ mol^−1^ NaCl over 45 cycles, less than half of typical reported values. These findings showcase NiCoP@NPC's superior SAC, ASAR, cycle stability, and low energy consumption. Analyzing the reasons, the extraordinary performance of NiCoP@NPC can be attributed to its unique structure, and strong synergistic effect among the components. First, due to the coupling of NPC and NiCoP, the diffusion path of Na^+^ is shortened, which reduces the charge transfer impedance and also promotes the electron transfer and the rapid diffusion of Na^+^.^[^ ^43^ ^]^ Second, the stable presence of Co^3+^/Co^2+^ and Ni^3+^/Ni^2+^ redox pairs in the complex excites the built‐in electric field effect and improves the reaction kinetics.^[^ ^35^ ^]^ In addition, the excellent redox activity of NiCoP effectively improves the electrochemical reaction efficiency during the electrochemical process.^[^ ^44^ ^]^ Finally, the structure of NiCoP@NPC has high mechanical strength, and the NiCoP NPs form strong chemical covalent bonds through surface confined growth, which prevents the stripping of the NiCoP NPs and thus ensures the structural integrity and cycling stability of the whole electrode during repeated charging and discharging processes.^[^ ^16^ ^]^

**Figure 5:**
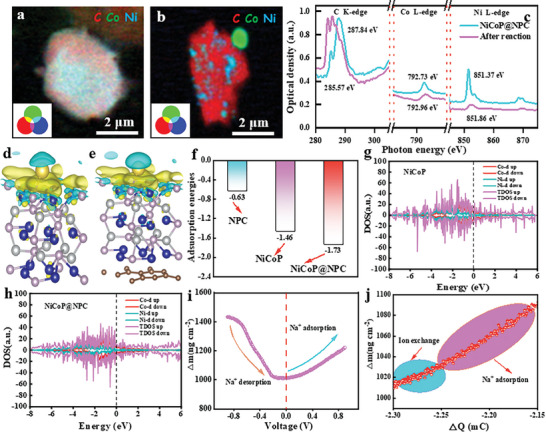
STXM chemical imaging of the original NiCoP@NPC a) and the NiCoP@NPC after reaction b), the green, blue, and red regions represent varying thicknesses, while pink identifies two hotspots of C/Co/Ni in the selected areas. XANES at C K‐edge, Co L‐edge, and Ni L‐edge extracted from different color regions in PCA analysis of the original NiCoP@NPC and the NiCoP@NPC after reaction c). Changes in electron density during the adsorption of Na in NiCoP d) and NiCoP@NPC e) are indicated by green and yellow colors, representing reductions and increases, respectively. f) Adsorption energy for different Na**^+^** adsorbed sites of NPC, NiCoP, NiCoP@ NPC. Electron density of states (DOS) for NiCoP g) and NiCoP@NPC h) surface adsorption of Na. i) The mass change of NiCoP@NPC at the scan rate of 30 mV s^−1^. j) NiCoP@NPC electrode mass versus charge passed during reduction at 30 mV s^−1^ from EQCM‐D.

STXM was applied to explore the mechanism of sample formation and after reaction. The STXM chemical images of the original NiCoP@NPC and the NiCoP@NPC after reaction are shown in **Figure** **5**a‐b. The stacked diagrams intuitively revealed that before the reaction, both the surface and the bulk phase of the yeast showed a homogeneous distribution of Co and Ni element as well as a higher concentration on the surface compared to that in the bulk phase, which may be closely related to the functional groups on the surface of the yeast (Figures S9 and S10a‐d, Supporting Information). This phenomenon further confirms that the yeast successfully realized the anchoring and transformation of Co and Ni element. After the reaction, the C surface of the yeast was basically exposed, thus indicating the redox reaction of the in situ‐anchored NiCoP NPs during the electrochemical reaction. Analysis of the C K‐edge in both samples revealed that the Carbon spectrum in the red area shifted to higher energies, which refers to stronger bonding with other elements.^[^ ^45^ ^]^ In addition, the surface area before NiCoP@NPC reaction C‐H, C‐OH, and C=O are present, while the features of C=C (285.1 eV) are only present in the surface area after desalination, the occurrence of a EDL during the desalination process implies the formation of a hydrated film, which alters the surface functional groups of the sample.^[^ ^46^ ^]^ Simultaneously, intensity changes in the green, red, and blue regions suggest covalent coupling between NPC and Co/Ni (Figure S11c‐d, Supporting Information). For the features of Co L‐edge at 792.73 eV and Ni L‐edge at 851.37 eV should be originated from the formed NiCoP NPs. In addition, the Co L‐edge and Ni L‐edge edges in the reacted NiCoP@NPC were also analyzed (Figure 5c). It can be clearly observed that there are many Co and Ni hot spots in the sample after the electrochemical reaction, which confirms that the Co and Ni elements in NiCoP@NPC after the reaction mainly exist in the form of Co and Ni (Figures S10e‐f and S11a‐d, Supporting Information). It can be further deduced that the desalination process occurs with the following reaction: NiCoP + 3Na^+^ + 3e^‐^–Co + Ni + Na_3_P.^[^ ^9a^ ^]^ As shown in Figure S11e‐f (Supporting Information), quantitative analysis of the elemental concentrations of the pristine NiCoP@NPC and the post‐reaction NiCoP@NPC reveals that the conversion of elemental Ni (10.9%) is higher than that of elemental Co in the process of Na^+^ storage and there is an aggregated difference in the distribution, which may be related to the redox activity of elemental Ni in the reaction system. In order to further analyze the reasons for the efficient desalination of the NiCoP@NPC, we conducted DFT calculations using the optimized theoretical calculation models of NPC, NiCoP, and NiCoP@NPC (Figure S12, Supporting Information). In the differential charge density, the yellow region indicates charge aggregation (electron gain), whereas the blue region indicates charge loss (electron loss). Upon comparing Figure 5d and Figure 5e, it is evident that the introduction of microbial carbon caused Na to lose more electrons, resulting in increased electron transfer with NiCoP and a stronger interaction force between the two. This enhanced interaction favored the adsorption of Na, making the adsorption of Na by NiCoP@NPC more stable compared to that of NPC and NiCoP. The results of the adsorption energy calculations (Figure 5f) revealed that the adsorption energy of Na^+^ by NiCoP@NPC (−1.73 eV) was the highest, followed by NiCoP (−1.46 eV), demonstrating that the incorporation of microbial carbon promoted the adsorption of Na^+^. Further analysis of the total density of states of the NiCoP@NPC and NiCoP NPs systems adsorbing Na revealed that the 3D orbital electrons of Co contributed significantly, followed by the contribution of the 3d orbital electrons of Ni. The density of states of NiCoP adsorbed Na exhibited sharper peaks across the energy intervals compared to that of NiCoP@NPC adsorbed Na, indicating relatively localized electrons in the former system. Additionally, the introduction of microbial carbon as a substrate improved the conductivity of NiCoP, along with lowering the electronic localization of NiCoP NPs after microbial loading, thereby facilitating electron transfer (Figure 5g‐h).

The process of embedding and removing Na^+^ from the NiCoP@NPC electrode was investigated throughEQCM‐D testing. As shown in Figure S13 (Supporting Information), the trends of frequency (Δf_3_/3) and dissipation (ΔD_3_) were the same during CV cycling at 0.02 and 0.03 V S^−1^. f_3_ and D_3_ basically recovered to their original values after one complete CV cycle, indicating that the mass change of Na^+^ in NiCoP@NPC is reversible and that the electrode changes during cycling were negligible^[^ ^47^ ^]^ (Figure S14, Supporting Information). Based on the EQCM‐D data, the mass change at the NiCoP@NPC electrode was quantitatively analyzed by the Sauerbrey equation^[^ ^48^ ^]^ (Figure 5i). Calculations showed that the mass of the NiCoP@NPC electrode gradually decreased when scanned at 0.02 V s^−1^ in the anodic interval, and the mass change reached a maximum of 421 ng cm^−2^ when scanned up to 0 V, returning to the starting value in subsequent cathodic scans, which was consistent with the trend of the frequency and dissipation factors. In addition, the analysis shows that Na^+^ storage process is divided into two phases: ion exchange and adsorption (Figure 5j). In conclusion, the EQCM‐D results not only confirm the reversible storage of Na^+^ in NiCoP@NPC electrodes, but also confirm that the proposed structural design strategy of NiCoP improves its structure and electrochemical cycling stability.

## 3 Conclusion

In summary, based on finite element simulation analysis, a microbial surface confined growth strategy was proposed, and a composite structure (NiCoP@NPC) with NPC and highly loaded NiCoP NPs was successfully designed and synthesized. The highly loaded and uniformly anchored NiCoP NPs significantly promoted Na^+^ diffusion and improved electrode kinetics, thus enhancing pseudocapacitance. Additionally, the NPC not only enhanced the electronic conductivity of NiCoP and provides intrinsic electrochemical capacity based on the EDL mechanism, but also improved the structural stability of the electrode by acting as a backbone to support the NiCoP NPs, and provides space to alleviate the stress concentration and volume expansion of the electrode. The structural advantages endowed NiCoP@NPC with a high Na^+^ storage capacity and a fast storage rate as well as a good cycling stability. In addition, DFT, STXM, and EQCM‐D tests confirmed that the yeast surface confined growth strategy significantly improved the Na^+^ storage capacity and cycling stability of the NiCoP electrode. This study offers valuable insights on the innovative design of composite structures using TMPs and conductive hollow materials for Na^+^ storage applications.

## Conflict of Interest

The authors declare no conflict of interest.

## Supporting information



Supporting Information

## Data Availability

The data that support the findings of this study are available from the corresponding author upon reasonable request.
