# Supplementary material for: Microbial Surface Confined Growth Strategy for the Synthesis of Highly Loaded NiCoP Nanoparticles with Hollow Derived Carbon Shells for Sodium Ion Capture
Source: Adv Sci (Weinh). 2024 Oct 28;12(1):2407616. doi: 10.1002/advs.202407616 (PMC11714236; doi:10.1002/advs.202407616)
Supplement: Supplementary file 1 — Supporting Information [file ADVS-12-2407616-s001.docx]

**Supporting Information**

**Microbial Surface Confined Growth Strategy for the Synthesis of Highly Loaded NiCoP Nanoparticles with Hollow Derived Carbon Shells for Sodium Ion Capture**

Jianhua Yuan^1,2,3^, Tianxiao Sun^4^, Jinfeng Chen^2^, Runhong Zhou^2^, Jianglin Cao^2*^, Fei Yu^5*^, Liqing Li^1*^, Xiumin Zhong^1^, Jie Ma^1,2,3*^

1 School of Chemistry and Chemical Engineering, Jiangxi University of Science and Technology, Ganzhou, 341000, P.R. China

2 Research Center for Environmental Functional Materials, College of Environmental Science and Engineering, Tongji University, 1239 Siping Road, Shanghai 200092, P.R. China

3 School of Civil Engineering, Kashi University, Kashi 844000, China

4 Helmholtz-Zentrum Berlin für Materialien und Energie GmbH, Hahn-Meitner-Platz 1, 14109 Berlin, Germany

5 College of Oceanography and Ecological Science, Shanghai Ocean University, Shanghai 201306, P.R. China

*Corresponding authors:

Jianglin Cao, E-mail: [jlcao@tongji.edu.cn](mailto:jlcao@tongji.edu.cn)

Fei Yu, E-mail: [fyu@vip.163.com](mailto:fyu@vip.163.com)

Liqing Li, Email: [liliqing79@126.com](mailto:liliqing79@126.com)

Jie Ma, E-mail: [jma@tongji.edu.cn](mailto:jma@tongji.edu.cn)

# 1. Materials and Methods

**1.1. Chemicals and Materials.** Cobalt nitrate hexahydrate, nickel nitrate hexahydrate and absolute ethyl alcohol (≥99.5%) and hydrochloric acid (36~38 vol.%) were obtained from Sinopharm Chemical Reagent Co., Ltd. (Shanghai, China). Nafion (5 wt.%) were purchased from Alfa Aesar (China) Chemical Co., Ltd. (Beijing, China). Poly (vinylidene fluoride) (average Mw. ~534,000) and N-methyl-2-pyrrolidone (≥98%) were gotten from Macklin Biochemical Co., Ltd. (Shanghai, China). Acetylene black was purchased from Aladdin Chemical Technology Co. Ltd., (Shanghai, China). All chemicals are used without any further purification.

**1.2. Preparation of NiCoP@NPC.** A 0.05 M solution of cobalt nitrate hexahydrate, 0.05 M solution of nickel nitrate hexahydrate and 50 ml deionized water were mixed in a conical flask. The prepared *Saccharomycete* Yeasts was transferred into the solution and the mixture placed in an oscillation incubator for 2 days. After completion of metal ion adsorption, the mixed solution was transferred to a 100 ml autoclave reactor and then hydrothermally heated at 180°C for 20 h. The resulting product was washed with ethanol solution and then freeze-dried at -80 °C under vacuum. The dried precursors were pyrolyzed at a temperature of 700°C for 2 h (5°C min^−1^) under nitrogen, the calcined samples were then washed and decontaminated with 2M hydrochloric acid and then dried and set aside. The final product is denoted as NiCoP@NPC. NPC was prepared by yeast cultivation without the addition of metal salts, and NiCoP was prepared by the addition of metal salts only without the addition of yeast cells. The preparation of both samples also included hydrothermal, cold-drying, and calcination processes.

**1.3. Electrode Preparation.** The active materials (NiCoP@NPC powder or activated carbon) were mixed with a conductive additive (acetylene black) and binder (polyvinylidene difluoride, PVDF) at a mass ratio of 8: 1: 1. Afterward, 1-methyl-2-pyrrolidinone (NMP) was added appropriately, and the mixture was stirred overnight to form a homogeneous and viscous paste. Then, electrodes were prepared by the doctor-blade method on graphite paper with a thickness of ca. 50 μm. Next, prepared electrodes were dried at 60°C under vacuum for 12 h to produce a slurry. The electrode’s mass was determined by the difference between pure graphite paper and dried electrode, and the electrode mass used for electrochemical desalination was about 10.0 mg.

**1.4. Electrochemical Characterization.** All electrochemical characterization, including cyclic voltammetry (CV), galvanostatic charging/discharging cycling (GCD), and electrochemical impedance spectroscopy (EIS), was conducted by the electrochemical station (CHI660D, Shanghai Chenhua Instruments Co.) in a three-electrode cell with 1 M NaCl as the electrolyte. A glassy carbon electrode (GCE, the diameter of glassy carbon is 3 mm.) coated with the as-prepared samples was used as the working electrode. A platinum sheet and Ag/AgCl were adopted as the counter and reference electrodes, respectively. The working electrodes were prepared according to similar method above.

Notably, the ratio of active materials, acetylene black, and polyvinylidene fluoride remains 8:1:1 (w: w: w), respectively. In addition, a certain volume of a 5 wt% Nafion solution was added into the slurry above. Cyclic voltammetry was swept between -0.90 V and +0.90 V under various scan rates (1-100 mV s^-1^), and GCD was tested under the same voltage window with different specific currents (0.10-0.50 A g^-1^). The EIS spectra were recorded over the frequency range of 10^5^ Hz to 10^-2^ Hz with an amplitude of 5 mV. Besides, CV and GCD were also carried out in a two-electrode cell with a working electrode and an oversized activated carbon powder (400 mesh, Macklin) electrode as the counter electrode in 1M NaCl.

The gravimetric specific capacitance based on CV curves (Cg, F g^-1^) was calculated

through the following equation:

$C_{g}=\frac{1}{2mv\Delta V}\int_{v_{1}}^{v_{2}} idV$ (1)

where m, v, V, and i are the mass of electrode (g), the potential scan rate (V s-1), the

potential window (V), and the current (A), respectively.

The power law relationship between the currents and scan rates of CV plots conforms the following formula:

$$i=av^{b} (2)$$

where i represents the current (A), v denotes the sweep speeds (mV s^-1^), a and b are constant values.

The capacitive contribution based on CV curves at different sweep speeds was calculated according to following equation:

$$i=k_{1}v+k_{2}v^{1/2} (3)$$

Where i(V) and v represent the total current at a given potential (A), the scan rate (V s^-1^), respectively. k_1_ and k_2_ are the constants. Of which, k_1_v and k_2_v^1/2^ denote the capacitive process and diffusion-controlled process, respectively.

**1.5. Electrochemical Desalination Performance.** The CDI performance was conducted in a continuous circulation system containing a power supply, data recording systems, a peristaltic pump, a conductivity meter, a CDI device, and the feed container. The CDI unit cell was washed by deionization water overnight before the CDI process. A certain concentration (1000 mg L^-1^) of the feed solution were pumped into the CDI unit cell. The volume and flow rate of the solution were kept at 40 mL and 25 mL min^-1^, respectively. Before desalination, the electrodes were rinsed by the feed solution without an external bias to achieve physisorption equilibrium and stabilize the solution conductivity. The specific adsorption capacity (SAC, mg g^-1^) was calculated by the following formula:

$$SAC=\frac{(C_{0}-C_{e})\times V}{m} (4)$$

where C_0_, C_e_, V, and m signify the initial and final NaCl concentrations (mg L^-1^), the volume of NaCl solution (L), and the total mass of two electrodes of CDI cell.

The time-average specific adsorption rate (SAR, mg g^-1^ min^-1^) was evaluated by the

following equation:

$$SAR=\frac{SAC}{t} (5)$$

where t is the charging time (min).

The energy-normalized adsorbed salt (ENAS, mg_NaCl_ J^-1^) was calculated according to following equation:

$$\text{ENAS= }\frac{\text{SAC}\text{×}\text{m}}{E_{in}}=\frac{SAC\times m}{V\int_{0}^{t} Idt} (6)$$

The transient specific adsorption rate was obtained through the differentiation of

desalination capacity with respect to time.

The energy consumption (EC, kJ mol^-1^) was obtained based on following formula:

$$EC=\frac{M_{NaCl}}{ENAS} \left( 7 \right)$$

where M_NaCl_ is the molar mass of NaCl (58.50 g mol^-1^).

**1.6. Density functional theory calculations.** The spin-polarized density functional theory (DFT) calculations were carried out in the Vienna ab initio simulation package (VASP) based on the plane-wave basis sets with the projector augmented-wave method. The exchange-correlation potential was treated by using a generalized gradient approximation (GGA) with the Perdew-Burke-Ernzerhof (PBE) parametrization. The energy cutoff was set to be 500 eV. The Brillouin-zone integration was sampled with a Γ-centered Monkhorst-Pack mesh ^6^ of 5 × 5 × 1 of NiCoP@NPC and NiCoP surface by VASPKIT^7^. The structures were fully relaxed until the maximum force on each atom was less than 0.03 eV/Å, and the energy convergent standard was 10^-5^ eV. The van der Waals correction of Grimme’s DFT-D3 model was also adopted ^8^. To avoid the periodic interactions for interface structures, a vacuum layer as large as 20 Å is used along the c direction normal to the interface. The adsorption energy of the first layer of Na atoms over understate was calculated by: E_ads_=E_Na+sub_-E_Na_-E_sub_, where the E_Na+sub_ was the total energy of Na adsorption on substrate, E_Na_ and E_sub_ were the total energy of Na atom and the substrate, respectively.

**1.7. Finite Element Analysis.** To investigate the role of the NiCoP@NPC structure in mitigating strain accumulation, the multiphysical field simulation software Comsol Multiphysics was used to simulate the displacement and stress of the electrode particle material after expansion. Solid mechanics was used as the main module to simulate the state during the electrode reaction. The post-expansion displacement was used to simulate the volume expansion of the electrode particles after the chemical reaction. In NiCoP and NiCoP@NPC electrode particles, Young's modulus of 206 and 88.50 GPa, Poisson’s ratio of 0.35 and 0.21, and densities of 8902 and 1750 kg m^-3^ were obtained for the particles and *Saccharomycete*, respectively.

**1.8 Electrochemical quartz crystal microbalance with dissipation monitoring (EQCM-D) experiments.** EQCM-D measurements was carried out on a quartz crystal microbalance equipped with an electrochemical module (QSense Explorer Electrochemistry, Biolin Scientific, Sweden). A typical electrode slurry was a mixture containing 80 wt.% HCl-NC, 10 wt.% carbon black, and 10 wt.% PVDF in NMP. After sonicating for 1 h, the slurry was coated on a quartz crystal disk with Au electrode (Biolin Scientific, Sweden).

**1.9. Material Characterization.** The formation process and surface morphology of NiCoP@NPC. were characterized by scanning electron microscopy (SEM, Hitachi Regulus8100) and transmission electron microscopy (TEM, JEOL-2010F). The crystal structure was analyzed using X-ray diffraction (XRD Bruker D8 Advance, Bruker AXS) operated at 40 mA and 45 kV with Cu-Kα radiation (λ =0:154nm, 5°/min). An X-ray photoelectron spectroscopy (XPS) analysis was carried out with a Kratos Axis Ultra DLD spectrometer using monochromatic Al Kα X-rays at a base pressure of 1×10^-7^ Pa. The peak energies were calibrated by placing the major C 1s peak at 284.8 eV. Raman spectra were recorded with a Renishaw in Via system using an Nd: YAG laser with an excitation wavelength of 532 nm. The specific surface area and pore size distribution were calculated from the adsorption/desorption isotherms of N_2_ at -196°C by the multipoint BET and BJH method using a BELSORP Max instrument (BEL), and the sample was degassed at 120°C for 6 h before the measurements.


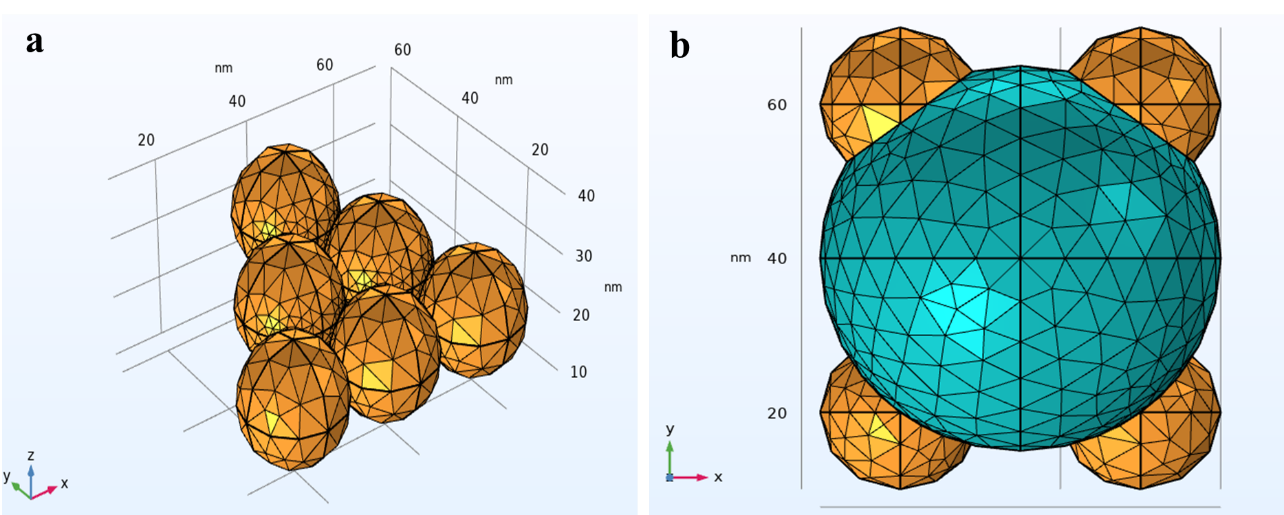


**Figure S1.** (a) Finite element model of individual NPs under particle accumulation and electrodes prepared after optimization strategy (b).

**
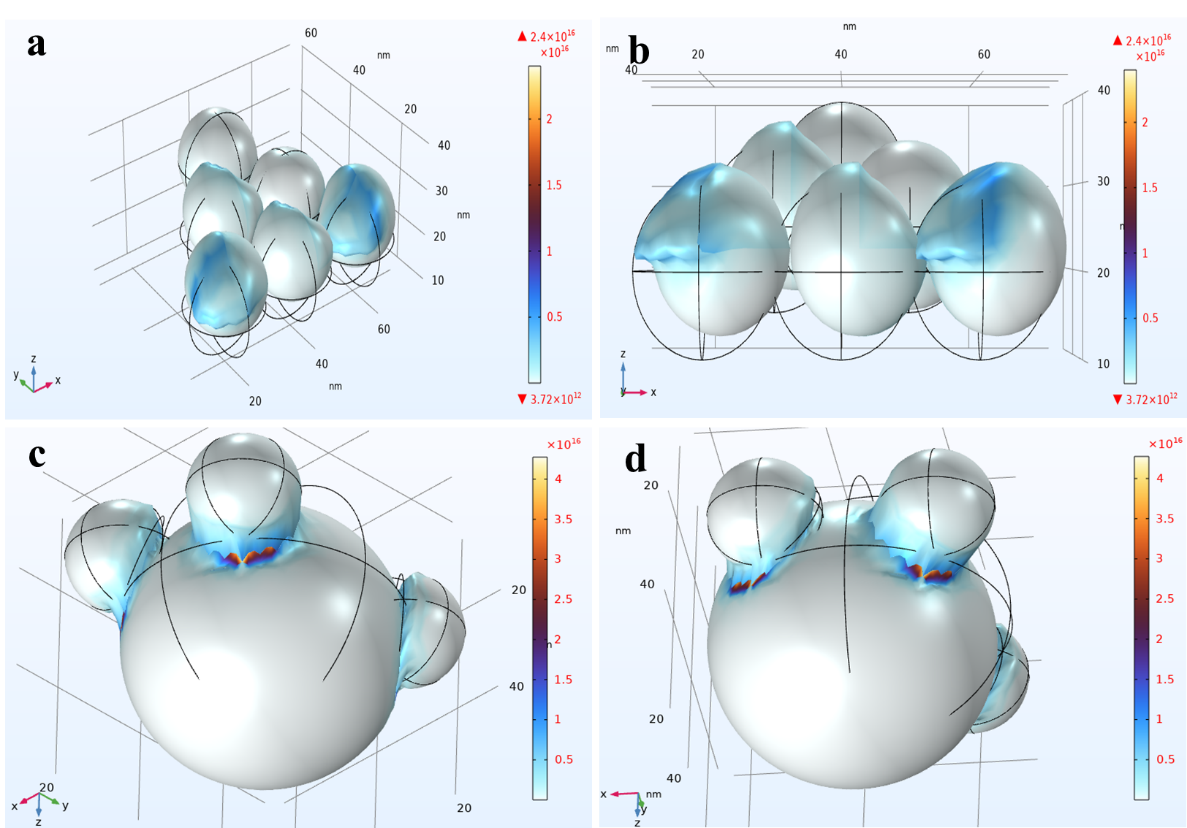
**

**Figure S2.** Finite element simulation of displacement and volume expansion of individual NPs under particle accumulation (a-b) and electrodes prepared after optimization strategy (c-d) in different perspectives.


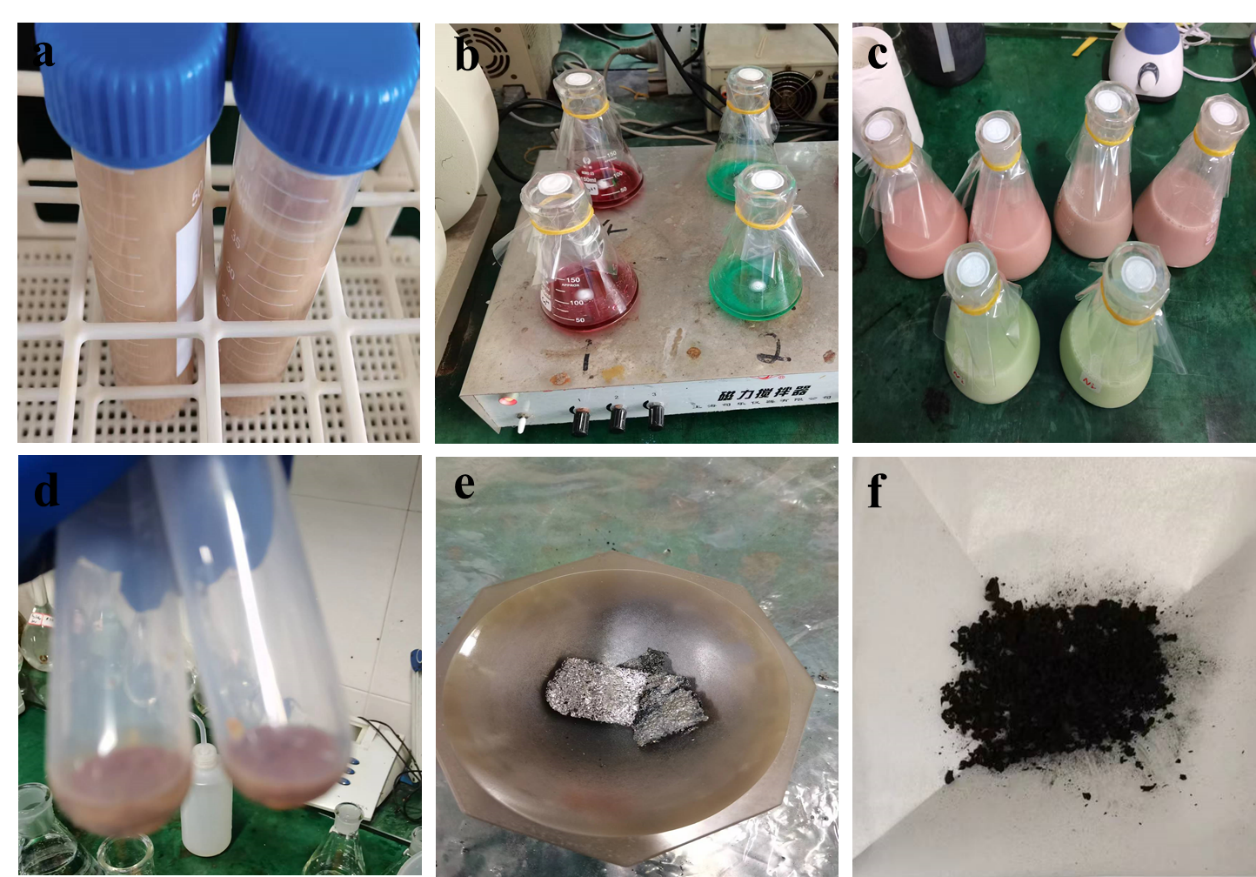


**Figure S3.** Photo of the process of *Saccharomycete* yeasts culture (a). (b-d) Adsorption of Co^2+^ and Ni^2+^ by *Saccharomycete* yeasts. (e-f) NiCoP@NPC after calcination treatment.


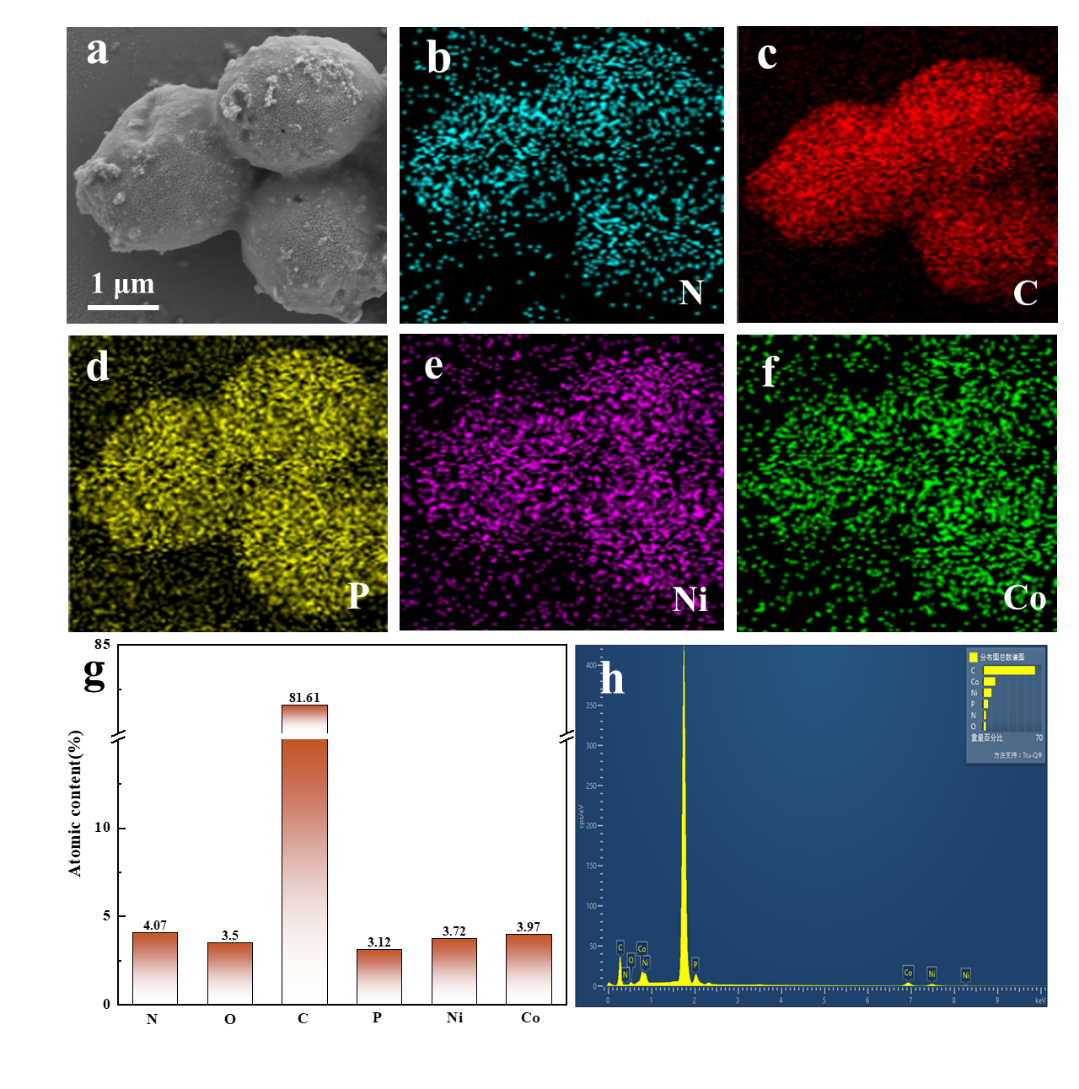


**Figure S4.** SEM images of NiCoP@NPC (a) and corresponding elemental mappings (b, c, e, f). (g) EDS of the NiCoP@NPC. (h) Atomic content of the NiCoP@NPC.


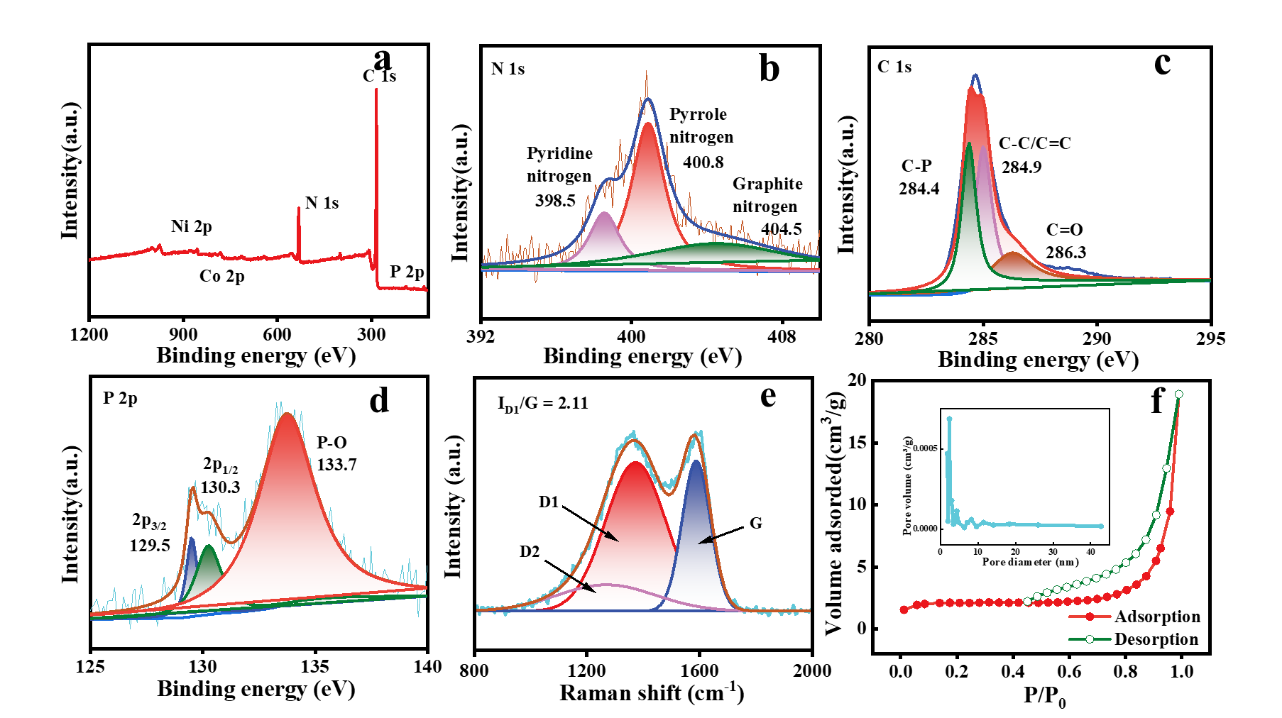


**Figure S5.** (a) XPS surface survey scan spectra of the NiCoP@NPC. High-resolution XPS spectra of N 1s (b), C 1s (c) and P 2p (d). (e) Raman spectra. (f) N_2_ adsorption/desorption isotherm curves and BJH pore size distribution of the NiCoP@NPC.

**Figure S6.** Thermogravimetric tests (TGA) of NiCoP@NPC.


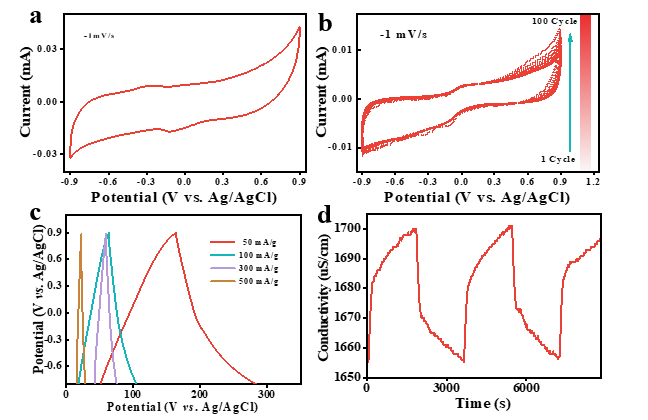


**Figure S7.** (a) Cyclic voltammograms for NiCoP@NPC at scan rate of 1 mV/s. (b) 100 cyclic CV curves at 1 mV/s. (c) GCD profiles for NiCoP@NPC at different density. (d) Conductivity changes over time. Desalination voltage is 1.6 V.

**
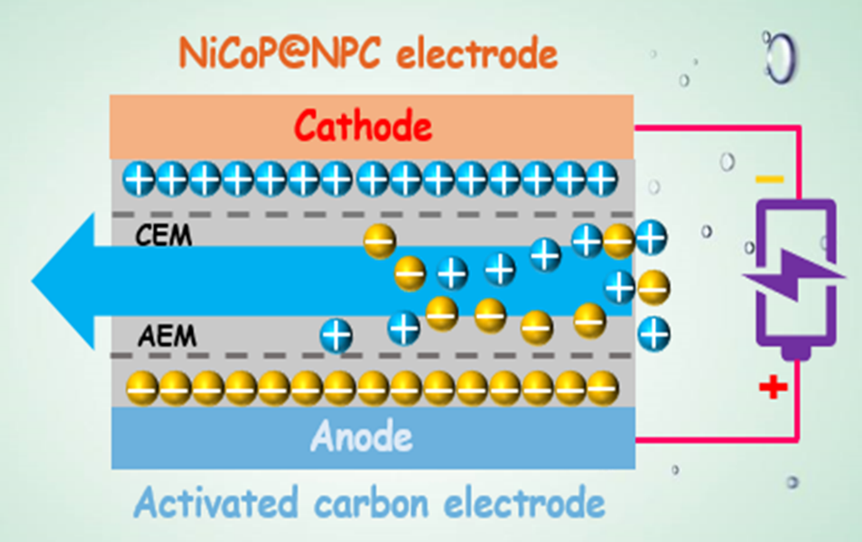
**

**Figure S8.** Schematic diagram of membrane-based capacitive deionization cell.


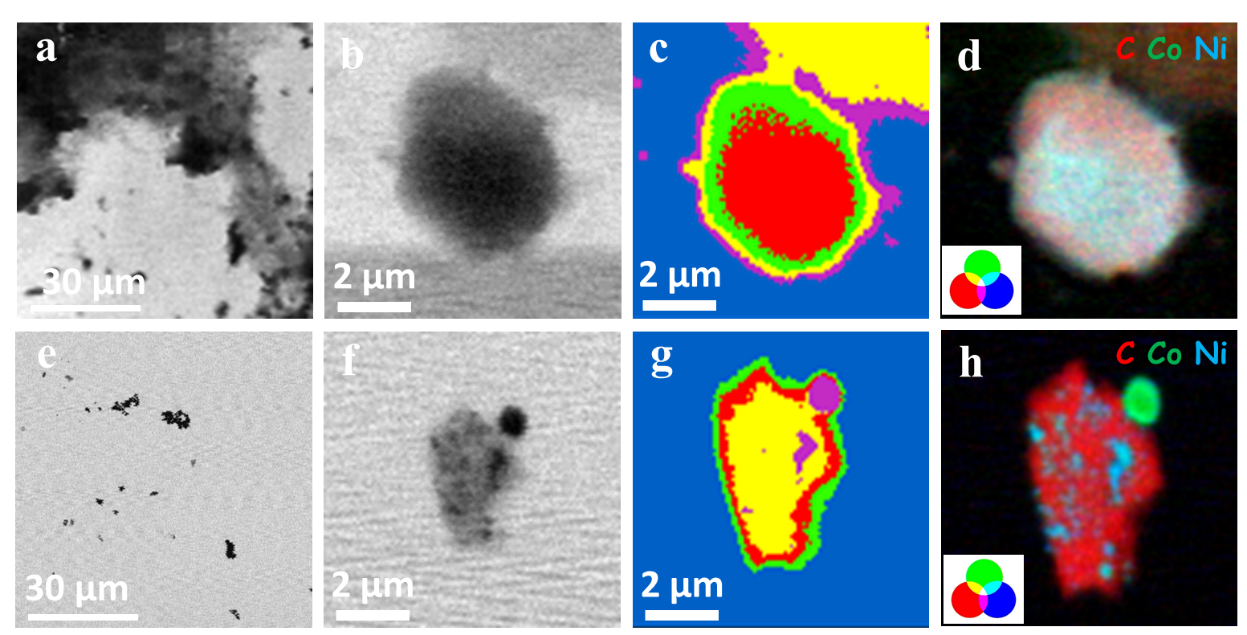


**Figure S9.** STXM Sample imaging of the original NiCoP@NPC (a-d) and the NiCoP@NPC after reaction (e-h).


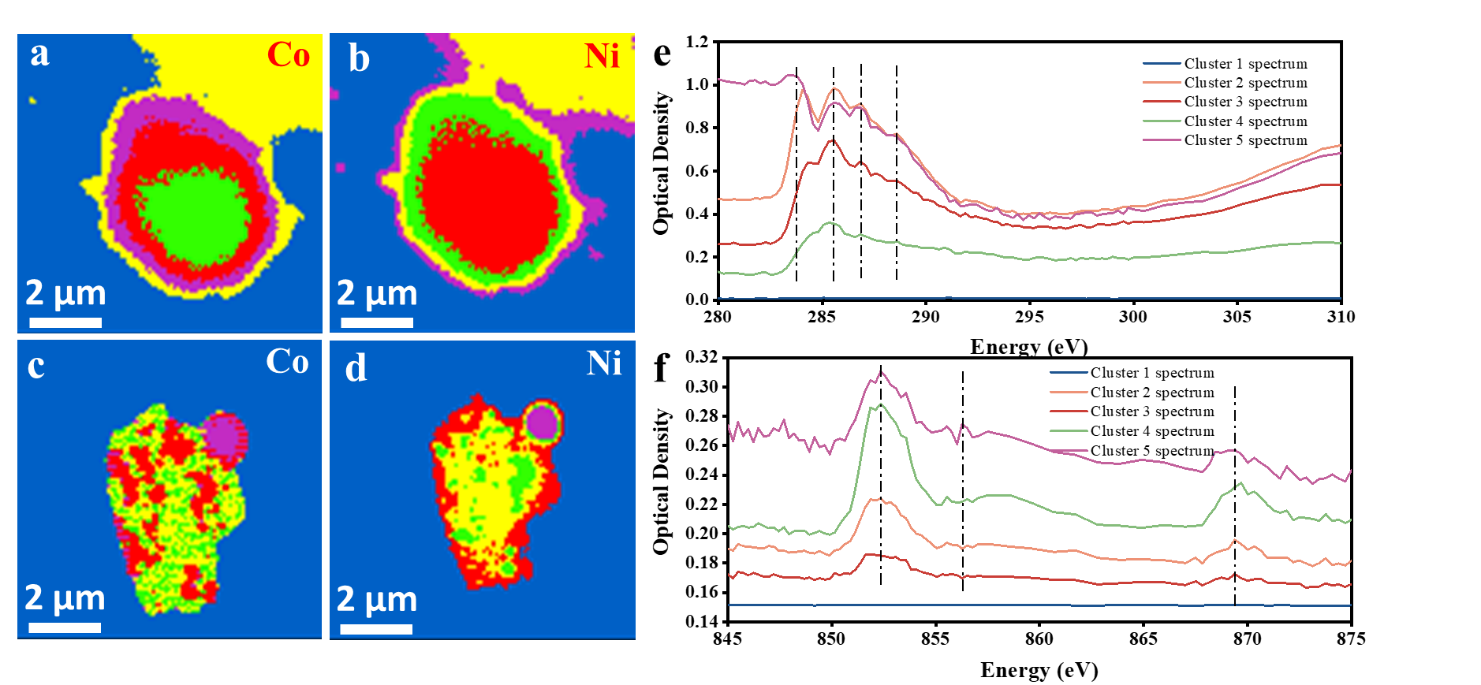


**Figure S10.** STXM chemical imaging of the original NiCoP@NPC (a: Co; b: Ni), and the NiCoP@NPC after reaction (c: Co; d: Ni). XANES at Co L-edge (e) and Ni L-edge (f) extracted from different color regions in PCA analysis of the NiCoP@NPC after reaction


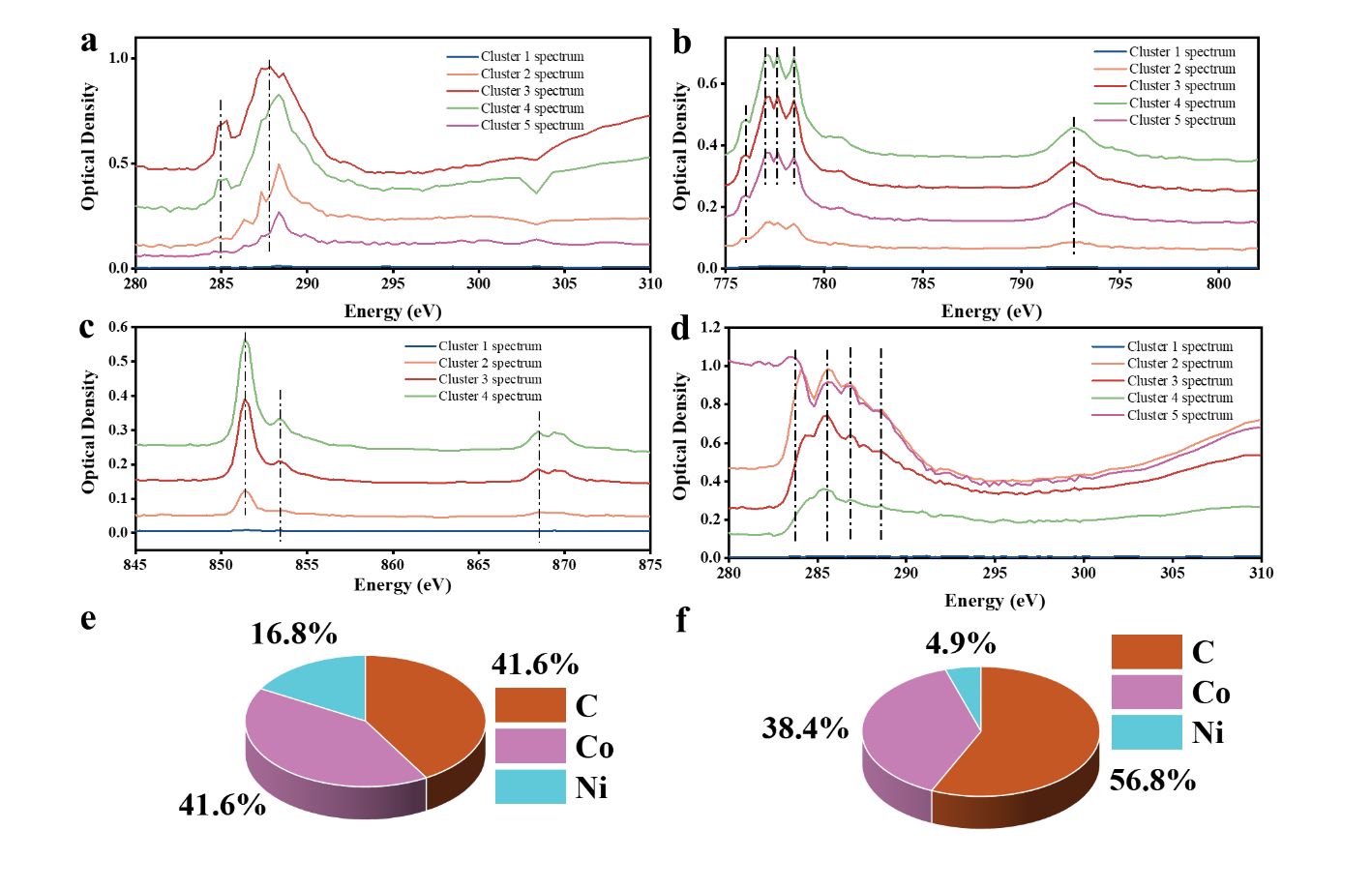


**Figure S11.** XANES at Co L-edge (a) and Ni L-edge (b) extracted from different color regions in PCA analysis of the original NiCoP@NPC. XANES at C K-edge extracted from different color regions in PCA analysis of the original NiCoP@NPC(c) and the NiCoP@NPC after reaction (d). Quantitative analysis of element concentration of the original NiCoP@NPC (e) and the NiCoP@NPC after reaction (f).


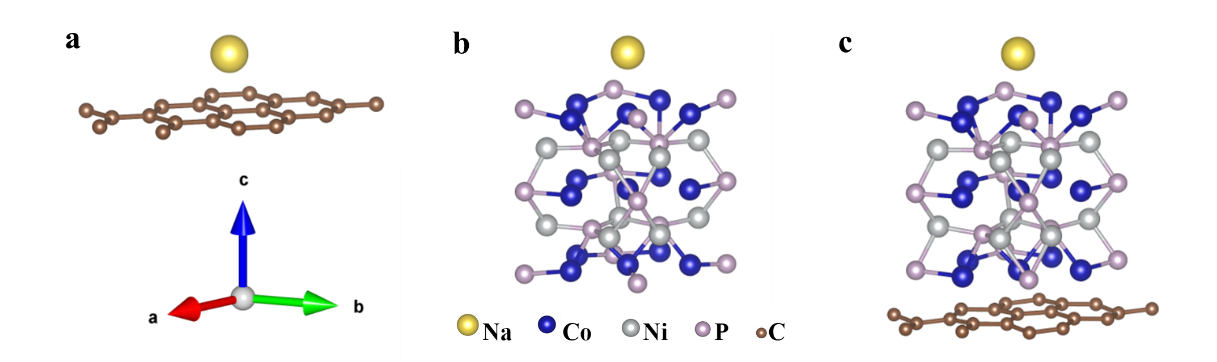


**Figure S12.** Adsorption model of NPC, NiCoP and NiCoP@NPC surface adsorption of Na.

**Figure S13.** △f_3_/3 and △D_3_ of NiCoP@NPC versus time at various scan rates during the 2nd CV cycle tested via EQCM.

**Figure S14.** △f_3_ and △D_3_ responses of NiCoP@NPC from EQCM-D during CV at the scan rate of 30 mV s^-1^ in 0.01 M NaCl. Three cycles were performed.

**Table S1.** Corresponding comparative results among different materials.

| Samples | NaCl concentration (mg L^-1^) | Desalination rate (mg g^-1^ min^-1^) | Adsorption capacity (mg g^-1^) | Ref. |
| --- | --- | --- | --- | --- |
| Cocoon | 1000 | 0.277 | 16.56 | [4] |
| Graphene/N-Doped mesoporous carbon | - | 0.035 | 18.4 | [5] |
| N/P co-doped 3D hierarchical carbon | - | 0.007 | 26.8 | [6] |
| Ag coated carbon | - | 0.078 | 15.6 | [7] |
| MoS_2_-graphene | 500 | 0.135 | 8.1 | [8] |
| D-MoS_2_ | 400 | 0.093 | 5.63 | [9] |
| Mo/CoS_2_@CNT | - | 0.626 | 37.55 | [10] |
| C@MoSP | 500 | 0.334 | 20.04 | [11] |
| Graphene@Co_3_O_4_ | 500 | 0.529 | 15.89 | [12] |
| CNT/ZnO | 400 | 0.187 | 22.55 | [13] |
| Na_2_FeP_2_O_7_ | 5844 | 0.081 | 30.2 | [14] |
| NiCoP@NPC | 1000 | 1.99 | 59.70 | This work |

**References**

[1] a) P. Hohenberg, W. Kohn, *Physical Review* **1964**, *136* (3B), B864, <https://doi.org/10.1103/PhysRev.136.B864>; b) W. Kohn, L. J. Sham, *Physical Review* **1965**, *140* (4A), A1133, <https://doi.org/10.1103/PhysRev.140.A1133>.

[2] a) G. Kresse, J. Furthmüller, *Physical review B* **1996**, *54* (16), 11169; b) P. E. Blöchl, *Physical Review B* **1994**, *50* (24), 17953, <https://doi.org/10.1103/PhysRevB.50.17953>.

[3] J. P. Perdew, K. Burke, M. Ernzerhof, *Physical Review Letters* **1996**, *77* (18), 3865.

[4] L. Zhang, Y. Liu, T. Lu, L. Pan, *Journal of Electroanalytical Chemistry* **2017**, *804*, 179, <https://doi.org/10.1016/j.jelechem.2017.09.062>.

[5] M. Zhou, X. W. Li, J. J. Cui, T. T. Liu, T. W. Cai, H. C. Zhang, S. Y. Guan, *International Journal of Electrochemical Science* **2012**, *7* (10), 9984.

[6] J. Han, L. Shi, T. Yan, J. Zhang, D. Zhang, *Environmental Science: Nano* **2018**, *5* (10), 2337, <https://doi.org/10.1039/c8en00652k>.

[7] H. Yoon, J. Lee, S. Kim, J. Yoon, *Desalination* **2017**, *422*, 42, <https://doi.org/10.1016/j.desal.2017.08.010>.

[8] J. Han, T. Yan, J. Shen, L. Shi, J. Zhang, D. Zhang, *Environ Sci Technol* **2019**, *53* (21), 12668, <https://doi.org/10.1021/acs.est.9b04274>.

[9] Z. Zhao, J. Zhao, Y. Sun, M. Ye, X. Wen, *Chemical Engineering Journal* **2023**, *458*, <https://doi.org/10.1016/j.cej.2023.141508>.

[10] *Chemical Engineering Journal* **2022**, *429*, <https://doi.org/10.1016/j.cej.2021.132582>.

[11] M. Zhao, Z. Zhao, X. Ma, J. Zhao, M. Ye, X. Wen, *Electrochimica Acta* **2021**, *387*, <https://doi.org/10.1016/j.electacta.2021.138494>.

[12] G. Divyapriya, K. K. Vijayakumar, I. Nambi, *Desalination* **2019**, *451*, 102, <https://doi.org/10.1016/j.desal.2018.03.023>.

[13] N. Arora, F. Banat, G. Bharath, E. Alhseinat, *Journal of Physics D: Applied Physics* **2019**, *52* (45), https://doi.org/10.1088/1361-6463/ab3967.

[14] S. Kim, J. Lee, C. Kim, J. Yoon, *Electrochimica Acta* **2016**, *203*, 265, <https://doi.org/10.1016/j.electacta.2016.04.056>.
